# Mobility of Zn and Cu in Bentonites: Implications for Environmental Remediation

**DOI:** 10.3390/ma17122957

**Published:** 2024-06-17

**Authors:** Edyta Nartowska, Anna Podlasek, Magdalena Daria Vaverková, Eugeniusz Koda, Aleksandra Jakimiuk, Robert Kowalik, Tomasz Kozłowski

**Affiliations:** 1 Faculty of Environmental Engineering, Geomatics and Renewable Energy, Kielce University of Technology, 25-314 Kielce, Poland; rkowalik@tu.kielce.pl (R.K.); tomkoz@tu.kielce.pl (T.K.); 2 Department of Revitalization and Architecture, Institute of Civil Engineering, Warsaw University of Life Sciences, Nowoursynowska 159, 02-776 Warsaw, Poland; anna_podlasek@sggw.edu.pl (A.P.); magdalena_vaverkova@sggw.edu.pl (M.D.V.); eugeniusz_koda@sggw.edu.pl (E.K.); aleksandra_jakimiuk@sggw.edu.pl (A.J.); 3 Department of Applied and Landscape Ecology, Faculty of AgriSciences, Mendel University in Brno, Zemědělská 1, 613 00 Brno, Czech Republic

**Keywords:** bentonite, mobility of toxic metals, physicochemical characterization of porous adsorbents, unfrozen water content, environmental protection

## Abstract

The aim of this study was to evaluate the mobility of copper (Cu) and zinc (Zn) and their impact on the properties of bentonites and unfrozen water content. Limited research in this area necessitates further analysis to prevent the negative effects of metal interactions on bentonite effectiveness. Tests involved American (SWy-3, Stx-1b) and Slovak (BSvk) bentonite samples with Zn or Cu ion exchange. Sequential extraction was performed using the Community Bureau of Reference (BCR) method. Elemental content was analyzed via inductively coupled plasma optical emission spectrometry (ICP-OES). Unfrozen water content was measured using nuclear magnetic resonance (^1^H-NMR) and differential scanning calorimetry (DSC). Results showed a significant influence of the main cation (Zn or Cu) on ion mobility, with toxic metal concentrations increasing mobility and decreasing residual fractions. Mobile Zn fractions increased with larger particle diameters, lower clay content, and shorter interplanar spacing, while the opposite was observed for Cu. Zn likely accumulated in larger clay pores, while Cu was immobilized in the bentonite complex. The stability of Zn or Cu ions increased with higher clay content or specific surface area. Residual Zn or Cu fractions were highest in uncontaminated bentonites with higher unfrozen water content, suggesting the potential formation of concentrated solutions in sub-zero temperatures, posing a threat to the clay–water environment, especially in cold regions.

## 1. Introduction

In recent years, there has been a growing interest in the topic of toxic metals, their mobility in the soil–water environment, methods of their immobilization, and removal from wastewater [1,2,3,4,5,6]. Metals accumulating in soil indirectly affect human and animal health through the food chain, dust, and contamination of surface and groundwater, potentially leading to serious damage [6,7,8]. Zinc (Zn) is an essential trace element supporting the immune system, but its excess can lead to immune system disorders and copper (Cu) deficiency; Cu aids enzymatic functions, but in excess, it causes liver and kidney damage [6,9]. The use of fertilizers containing high concentrations of Cu and Zn, industrial activities, and flotation tailings storage are the most common sources of these metals [10,11,12]. Both Cu and Zn are trace elements that play vital roles as essential nutrients for plants. However, their excessive accumulation in the soil can lead to harmful effects. For instance, elevated levels of these metals can decrease plants’ nutrient uptake capacity and reduce the population of soil microorganisms. Consequently, this can disrupt vital soil biological processes, including organic matter decomposition and the nitrogen cycle [12,13,14]. The heavy metals nickel (Ni), lead (Pb), and chromium (Cr) pose a significant concern for water and soil environments. Their leaching from municipal or industrial waste can result in soil contamination and toxicity to organisms inhabiting it. Moreover, Cr and Pb can also undergo bioaccumulation in living organisms [15]. Liao et al. [16] reported that soil pollution by Ni and Cu is an extremely serious problem. Once in the soil, these metals move within the soil and are not degraded by microorganisms. They are susceptible to transformation into various chemical forms such as dissolved ions, adsorbed forms, exchangeable forms, and complexes with organic matter through processes such as dissolution, adsorption, coagulation, and complexation [17]. 

Interactions of potentially toxic metals with soils are a complex and dynamic process that is still not fully understood. Feng et al. [18] and Li et al. [2] believe that significant influences on these processes are interactions of metals with microorganisms, organic matter, and clay minerals. The main components of clay minerals are silicate minerals and oxides, which affect the binding and migration of metals in soils [19]. Clay minerals, such as montmorillonite, which is the main component of bentonites, are often used to improve soil quality for various applications due to their high specific surface area, ion exchange capacity, and strong stability. One of these applications is their use as a mineral barrier in waste landfills, including hazardous ones [3,20,21,22]. Bentonites in contact with various chemical substances, including ions of potentially toxic metals, are not inert substances and may undergo degradation in the interactions due to clay dissolution, cation exchange between layers, or ion competition [3,23]. As a result, the engineering properties might change, which were initially meant to keep the facility and the environment safe [24]. In cold regions, the effects of toxic metal interactions in bentonites can be more hazardous. The remaining liquid phase in frozen soils, known as unfrozen water, forms a concentrated solution, whose ions actively interact with the surface ions of minerals, often leading to changes in its volume [25,26]. Therefore, assessing the mobility of potentially toxic metals in the clay–water environment, especially in bentonites, is an important engineering and environmental issue. Understanding the interactions of ions of potentially toxic metals in bentonites can help explain their migration mechanisms, which may minimize the risk of their spreading into the soil–water system through appropriate protective measures in the future.

To study the dynamics of changes occurring in the soil–water system contaminated with ions of potentially toxic metals, it is important to determine not only the total concentration of metals present in the soil, but also their chemical form, by which they become mobile. Sequential extraction, which separates metals into pools with different tendencies to migrate in the soil medium, should be the basis of studies in this field [2,27,28]. One of the generally accepted and most widely used sequential extraction methods, next to the Tessier method, is the BCR technique [28,29]. This method consists of four steps, each using chemical extraction solutions of increasing chemical aggressiveness. This allows the division of metals into fractions (F): F_I_, exchangeable and associated with carbonates; F_II_, associated with Fe and Mn oxides; F_III_, associated with organic matter and sulfides; and F_IV_, associated with primary and secondary minerals. Metals in fractions I and II are generally considered to pose the greatest threat to the aquatic soil environment, whereas those in fraction IV are considered unavailable [28]. A literature review has established that certain factors can influence the mobility of toxic metals in clayey soils, many of which are directly related to changes in the soil’s physicochemical properties due to chemical interactions with the solution. However, these mechanisms are highly complex, and the situation is challenging because metal ions interact with substances dissolved in the pore solution during migration, which can affect the efficiency of metal migration [30]. Various factors influence the mobility of toxic metals in clayey soils, including the anthropogenic origin of metals, bentonite quality, metal concentration, temperature, presence of organic matter, proportion of clay minerals, and pH [24,31,32,33]. Anthropogenic contamination of soils with cadmium (Cd), Zn, and Pb increases their mobility [24]. Dutta et al. [34] and Nartowska et al. [35] suggest that at low concentrations of metal salts in bentonites, bentonite quality affects clay permeability, while at higher concentrations, it becomes the dominant factor. He et al. [36] propose that, in low-permeability soils, solute transport results from the interaction of adsorption and diffusion forces, with solution molarity playing a significant role. For instance, increased Zn retention was observed with increasing ionic strength of the solution. Nartowska et al. [26] observed lower unfrozen water content in bentonites contaminated with Zn or Cu ions compared to uncontaminated bentonites. They also identified statistically significant relationships between unfrozen water content and the clay fraction in bentonites contaminated with Zn or Cu. However, under positive temperature conditions, the behavior of bentonites contaminated with Cu ions is more influenced by changes in their structural parameters, including specific surface area [35,37], while in bentonites contaminated with Zn ions, it is related to changes in their granulometric parameters [24]. Huang et al. [38] found that the size and characteristics of soil aggregates significantly affect the mobility and bioavailability of heavy metals in soils. Some authors suggest that the content of Cu, Pb, and Zn increases with decreasing soil particle size, while others attribute this relationship to the type of metal [2]. The presence of clay minerals and organic matter generally promotes metal immobilization and reduces their mobility [39]. The potential of hydrogen (pH) is cited as one of the most significant parameters affecting the total concentration of metals in soils [33,40] and is crucial for assessing metal mobility [41,42]. However, the observed variability in metal mobility cannot be solely explained by changes in pH [31]. Variations in metal adsorption capacity can be attributed to acidic modification of clay surface properties [43]. In turn, potentially toxic metals can exist as particles adsorbed onto clay surfaces, highlighting their undeniable relationship with the physicochemical properties of the soil–water system [44,45].

The characteristics of the migration and transformation of contaminants in altered soils require further study [30]. There is a lack of research in the literature regarding the following issues:

(i) The mobility of potentially toxic metals in homogeneous (model) clays devoid of organic matter, or in bentonites, was evaluated. Studies on source clays will facilitate the interpretation of the processes responsible for the mobility of potentially toxic metal ions so that the results can be more easily related to natural conditions. In addition, it may not be possible to identify the mechanisms governing sorption in systems with many metals in the clay [46];
(ii) The relationships between the mobility of potentially toxic metals and the physicochemical properties of soils can be interpreted. The authors emphasized that this is especially important in clay soils, where a significant portion of the metals may be bound to the clay surface. Li et al. [2] emphasized that the interactions between potentially toxic metal ions and clay minerals require further research. Ma et al. [19] emphasized that understanding the mechanisms and suitability of different types of clay minerals in immobilizing toxic metals is crucial to achieving remediation goals;
(iii) The mobility of toxic metals, in the context of their physicochemical properties at subzero temperatures, was evaluated. Understanding the interactions of these processes under freezing conditions is important because the concentrations of mineral salts and other substances are highest in very-cold winters [45]. Because water can be a carrier for the migration of potentially toxic metals in cold regions, it is important to know the relationship between these factors to take appropriate steps to minimize the risk. It is particularly important to understand the relationships between the chemical fractions of metals that determine their mobility and changes in unfrozen water content. To date, no such studies have been conducted.

Thus, the main objective of this study was to evaluate the mobility of potentially toxic metals, Zn or Cu, in bentonites using the BCR procedure in the temperature range 20 °C to −32 °C. Specific objectives included the following: (i) determination of the total concentration of potentially toxic metals (Zn, Cu, Cr, Ni, and Pb) and their contribution to chemical fractions determined via the BCR procedure in uncontaminated bentonites and in bentonites contaminated with Zn or Cu ions of different origins; (ii) determination of the relationship between the total concentration of Zn or Cu, microstructural properties, physicochemical properties of bentonites, and the proportion of chemical fractions of these metals in bentonites contaminated with Zn or Cu ions; and (iii) determination of the relationship between Zn or Cu chemical fractions, microstructural and physicochemical properties, and the content of unfrozen water in bentonites contaminated with Zn or Cu ions at temperatures below 0 °C. Achieving the objectives allowed for a comprehensive analysis of the impact of contaminating bentonites with Zn or Cu ions on their physicochemical properties, which is applicable in both environmental protection and materials engineering. The urgency of the research hypothesis stems from the need to understand the mobility of these metals in bentonites under various environmental conditions, including low temperatures, which is crucial for assessing environmental hazards in cold climate regions and for effective waste landfill management. The main innovations of the present work are as follows: (i) the use of model bentonites, whose main exchangeable cation is Zn or Cu, to analyze the mobility of potentially toxic metals. Because bentonites are widely used worldwide as adsorbents for toxic metals, knowledge of their mobility in these clays is of particular importance; (ii) exploration of the relationship between the chemical fractions of metals and properties of bentonites. This knowledge is also useful when planning remedial works at waste landfills. An example of an engineering solution is the study by Koda et al. [20,21,22], who designed a cut-off bentonite barrier for groundwater protection at the sanitary landfill in Radiowo, Poland; and (iii) capturing the relationships between the physicochemical parameters of bentonites and the unfrozen water content at subzero temperatures. This knowledge is particularly important in cold regions, and the global literature on this subject is still limited.

## 2. Materials and Methods

### 2.1. Materials

Three model clays were used to assess the mobility of potentially toxic metals in bentonites: SWy-3 (Na-Ca bentonite from Crook County, WY, USA), STx-1b (Ca bentonite from Gonzales County, TX, USA), and BSvk (Ca bentonite from Jelsovy Potok, Slovakia). The American bentonites were obtained from the Source Clays Repository of the Clay Mineral Society, while the Slovak bentonite was obtained from ZGM Zębiec S. A., Starachowice, Poland. Each of the three model bentonites was tested in its natural form and previously prepared homoionic forms (Zn or Cu). Nine homoionic forms of bentonites were obtained, designated as follows: SWy-3 1M Cu, STx-1b 1M Cu, and BSvk 1M Cu, copper forms of bentonites formed via saturation with 1 mol/dm^3^ copper (II) chloride solutions; BSvk 0.1M Cu, BSvk 0.25M Cu, and BSvk 0.5M Cu, copper forms of Slovak bentonite formed via saturation with copper (II) chloride solutions (Chempur, Poland, CAS No. 10125-13-0) of 0.1–0.5 mol/dm^3^; and SWy-3 1M Zn, STx-1b 1M Zn, and BSvk 1M Zn, zinc forms of bentonites formed via saturation with zinc (II) chloride (Chempur, Piekary Śląskie, Poland, CAS No. 7646-85-7) solutions of 1 mol/dm^3^. A significant disparity in the content of mobile Cu fractions in bentonites of different origins was observed, leading to a study of the effect of Cu ion concentration on the proportions of copper BCR fractions in selected BSvk bentonite at various concentrations.

In the BCR studies, the following reagents (Chempur, Poland) with their respective CAS numbers were used:

(i) CH_3_COOH 0.11 mol/dm^3^ (CAS No. 64-19-7);
(ii) NH_2_OH·HCl 0.5 mol/dm^3^ (CAS No. 5470-11-1);
(iii) H_2_O_2_/CH_3_COONH_4_ 8.8 mol/dm^3^ (CAS Nos. 7722-84-1 and 631-61-8);
(iv) HNO_3_ and HCl 1 mol/dm^3^ (CAS Nos. 7697-37-2 and 7647-01-0).

**Figure 1:**
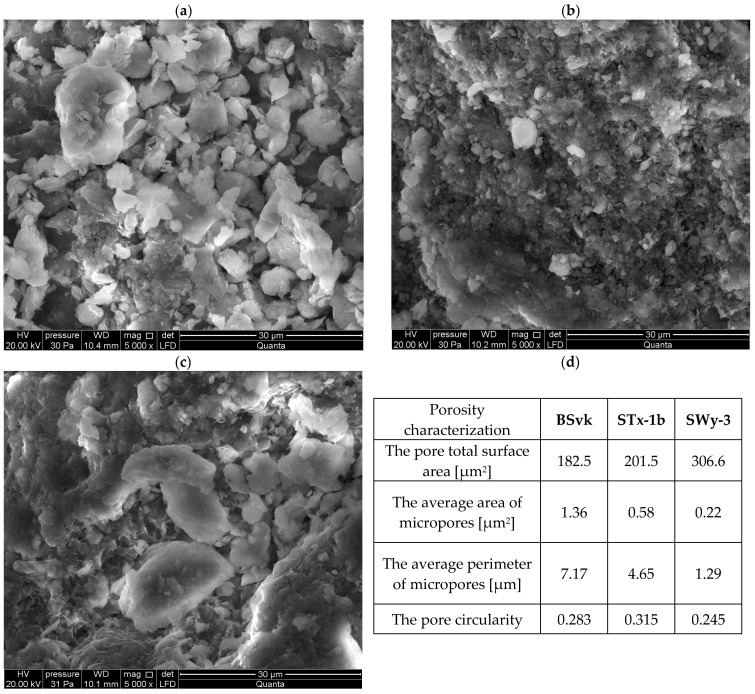
SEM images and porosity characterization for BSvk, STx-1b, and SWy-3 bentonites. (**a**) BSvk bentonite. (**b**) STx-1b bentonite. (**c**) SWy-3 bentonite. (**d**) Porosity characterization using SEM–NIA method acc. to Nartowska et al. [35].

The source clays along with an assessment of their porosity were shown in SEM microphotographs in Figure 1. Source bentonites have a cellular microstructure.

**Figure 2:**
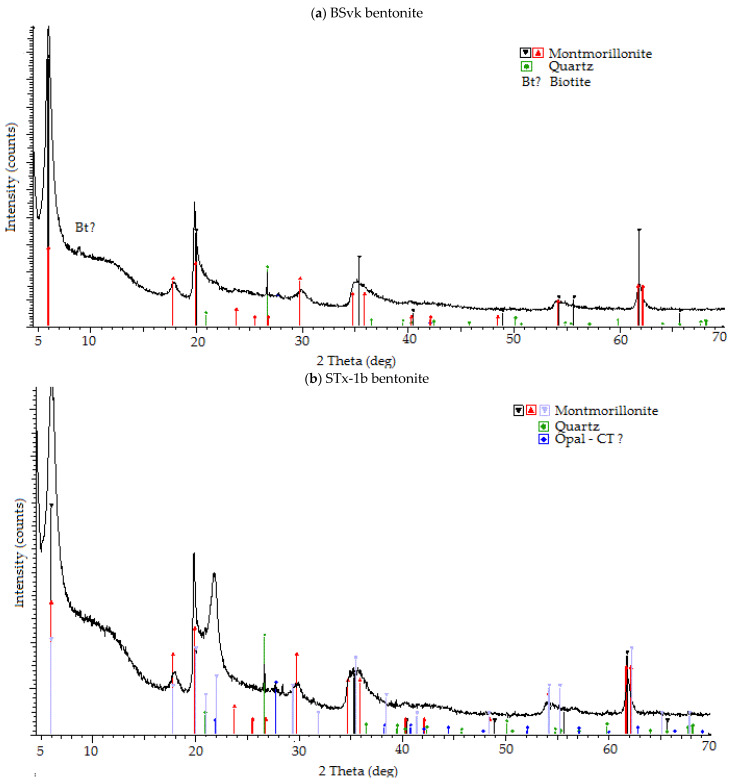

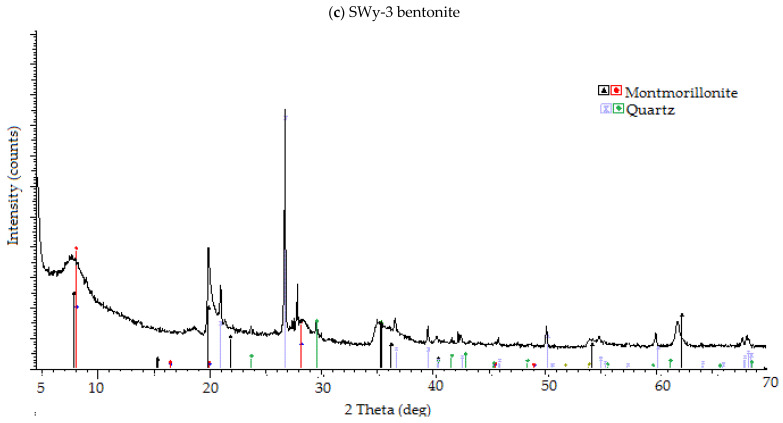
XRD for BSvk, STx-1b, and SWy-3 bentonites.

Each bentonite contained at least 70% montmorillonite in its mineral composition, in addition to silica minerals and biotite, and in the case of bentonites contaminated with Cu 0.1–0.5 M ions, 3–6% of atacamite (copper hydroxychloride) minerals were observed [47]. Source bentonites, according to the diffractograms (Figure 2), contained the following: 70% montmorillonite, 30% quartz (SWy-3); 75% montmorillonite, 20% opal-CT, 5% quartz (STx-1b); and 92% smectite, 5% quartz and 3% biotite (BSvk) [47]. The results are nearly consistent with the data presented in the literature [48,49].

#### Preparation of Homoionic Forms

Bentonite (50 g) was flooded in buckets with 10 liters of zinc (II) chloride solutions of 1 mol/dm^3^ and copper (II) chloride solutions at concentrations of 0.1–0.25–0.5–1 mol/dm^3^ to obtain homoionic forms of clay. Flooding was performed at intervals of 48 h, after which the solution was decanted from the sediment and the flooding process was repeated with a new portion of the solution. The operation was repeated three times. Subsequently, the remaining suspension was transferred to chloride ion-permeable membranes, which were immersed in a bucket with a constant circulation of distilled water. The water was changed every 48 h for approximately 35 days. Excess chlorine was flushed out until the characteristic reaction in the water to Cl^−^ ions (from 0.1 mol/dm^3^ AgNO_3_, Chempur, Poland) disappeared. The monoionic bentonite samples were air-dried and sequentially transferred to sealed jars. The details of the methodology are included in the authors’ previous work [26]. In addition, the absence of free chloride ions in all 12 clays was confirmed with analytical methods. Solutions consisting of 2 g clay and 20 ml of distilled water were shaken for 24 h on a rotary shaker at room temperature. The samples were successively filtered through Whatman blotting paper, grade 1.6 µm, 50 mm diameter circle (Maidstone, Kent, UK). The prepared solutions were titrated with silver nitrate in the presence of chromate as an indicator (PN-ISO 9297:1994 [50]).

### 2.2. Methods 

#### 2.2.1. Determination of the Content of Potentially Toxic Metals

A sequential extraction procedure was used to fractionate the metals according to their mobility, providing insight into the ionic dynamics of potentially toxic metals in bentonites. This is crucial because the hazard of a metal depends on its specific form. Understanding the toxic and chemical forms of metals is often more important than knowing their total content, because their bioavailability is closely determined by their mobility [28,51]. Zimmermann and Weindrof [29] confirmed that the total content of elements in soils does not correspond to the processes in which they participate in the environment, and that only studies using sequential extraction can provide a basis for investigating the dynamics of changes in the soil–water environment.

This study used a four-step BCR sequential extraction with a modification introduced by European researchers [52]. This method is considered one of the widely accepted methods in soil chemistry, the results of which are easy to reproduce [29]. The residual fraction was mineralized using aqua regia. Potentially toxic metals were separated into individual mobility fractions that showed different tendencies to migrate in the clay medium. To determine the subsequent fractions, the test clay was dissolved in a reagent specific to that fraction [53]. The mixtures were then centrifuged to separate the solid phase from the solution that contained the extracted metals. The contents of potentially toxic metals (Cu, Zn, Cr, Ni, and Pb) in the extracts were determined using a Perkin Elmer Optima 8000 inductively coupled plasma ICP–OES optical emission spectrometer (PerkinElmer, Waltham, MA, USA). The BCR procedure involves the following steps (Table 1).

In all the bentonites tested, the total concentration of metals was additionally determined as an independent test, which is useful to assess the recovery of metals from individual BCR fractions and to establish the relationship between the total concentration of Zn or Cu and the proportion of their mobile and immobile fractions. The procedure was the same as that in step 4. The results of these analyses are summarized in Table A1 and Table A2 in the Appendix A.

#### 2.2.2. Determination of the Physicochemical Properties

The granulometric composition was analyzed using a HELOS/BF SUCELL laser diffractometer from Sympatec GmbH (Clausthal-Zellerfeld, Germany), equipped with a ‘wet’ test attachment, according to the ISO 13320:2020 standard [54] and Sympatec guidelines [55]. The clay samples were prepared 48 h prior to testing by processing into clay pastes. Immediately prior to testing, 3 g of clay paste were mixed with 50 ml of distilled water. A sample of a few milliliters was taken for analysis to ensure that the optical concentration of particles in the test chamber did not exceed 25%. A reference measurement was made before each new measurement [32]. The diameters d_10_ and d_32_ were obtained from the generated granulometric composition curves. These values denote grain diameters, the mass proportion of which, together with smaller grains in a given clay, is 10% and 32%, respectively. Effective diameters are used in many soil filtration models, so they may prove useful in analyzing the dynamics of potentially toxic metals in porous clays such as bentonites [24]. Additionally, the d_32_ diameter, known as the Sauter diameter, is a commonly used measure in fluid dynamics as the mean surface volume diameter of a particle. It is thought to characterize many important processes, including chemical processes [56].

The specific surface area was determined, which is useful in the analysis of clay–water systems, using the water vapor sorption (WST) method [26]. The specific surface area was determined according to Equation (1):

(1) $$S=6\cdot \left(w_{50}\cdot 5.85\right)$$

where w_50_ is the relative water vapor pressure (*p*/*p*_0_ = 0.50) in a desiccator over a saturated solution of magnesium nitrate (V) for 10 days.

The mineral composition and the interpacket distances in the bentonites, in the plane parallel to the sample surface (d_001_), were determined based on X-ray diffractograms. Changes in inter-packet distances serve as a useful tool for assessing the metal dynamics, ion exchange, and adsorption properties of bentonites [57]. For this study, a Bruker D8 Advance X-ray diffractometer with a Johansson-type monochromator was employed to determine the CuKα1 radiation (λ = 1.5406 Å) with a LynxEye position-sensitive detector (Bruker devices, Berlin, Germany). The measurements were conducted in the 2θ range from 4.51° to 70° with 0.02° steps. The applied voltage was 3.540 kV with a current of 530 mA [57].

#### 2.2.3. Determination of the Changes in Unfrozen Water Content

In soils, there is a fraction of liquid water that does not freeze even at very-low subzero temperatures (e.g., up to 20% of this fraction can be expected in bentonites at T = −30 °C). This is referred to as unfrozen water content. Its amount depends on the temperature, type of soil, physical and chemical parameters, and soil chemistry.

For the analyses, the authors used the results of changes in the unfrozen water content of bentonites published in their previous works [26,47]. The mean unfrozen water content and standard deviation are summarized in Table A3. To study the changes in unfrozen water content in the Zn-bentonites, the technique of ^1^H NMR (nuclear magnetic resonance) was employed. This study was conducted with a minispec mq20 spectrometer (Bruker, Berlin, Germany). The content of unfrozen water at particular freezing temperatures was determined by changes in the intensity of the free induction decay (FID) signal, which is directly proportional to the number of ^1^H hydrogen nuclei and thus to the volume of water and ice in the clay sample. Unfrozen water was calculated according to Equation (2). The highest FID signal value for a given sample above 0 °C was determined according to Nartowska et al. [58]:

(2) $$w_{u}=\frac{{\mathrm{SI}}_{\mathrm{FID}}(T<0{}^{\circ}C)\cdot w_{n}}{{\mathrm{SI}}_{\mathrm{FID}}}$$

where w_u_ is the unfrozen water content (% of clay dry weight), SI_FID_ is the signal intensity of the free inductive decay at a given temperature (in this study, the highest signal value for a given sample above 0 °C was determined [58]), and w_n_ is the natural water content of the clay (% clay dry matter).

Based on preliminary studies of changes in unfrozen water content in Cu bentonites using the ^1^H NMR technique [26,58], it was suspected that the determination results might be affected by ion hydration, which requires further research [58]. Therefore, DSC was used to evaluate the changes in unfrozen water content in the Cu bentonites. The tests were performed using a DSC Q200 instrument (New Castle, DE, USA, TA Instruments) with a liquid nitrogen cooling system (RSC 90). The samples were cooled at a scan rate of 2.5 K/min to −90 °C, followed by heating at a scan rate of 5 K/min to 20 °C. Due to the possible phenomenon of overcooling, the heating thermograms were analyzed. The unfrozen water content at a given temperature was calculated using Equation (3):

(3) $$w_{u}\left(T_{i}\right)=w-\sum_{j=i}^{n}\frac{100\cdot q\left(T_{j}\right)\cdot \Delta T_{j}}{L\left(T_{j}\right)\cdot m_{s}}$$

where *w_u_*(*T_i_*) is the unfrozen water content at temperature *T_i_* as a percentage of dry mass, *w* is the water content expressed as a percentage of dry mass, *m_s_* is the mass of dry clay in the sample (g), and *L*(*T_j_*) is the latent heat of fusion of ice at temperature *T_j_*, calculated according to the empirical equation *L*(*T*) = 7.3*T* + 334.

#### 2.2.4. Data Quality Control

The methods were based on the ISO/IEC 17025 guidelines [59]. Source clays with one main exchangeable cation, which determines the physicochemical properties of bentonites, were used in this study. Bentonites are materials with a grain size of d < 63 μm, which eliminates the possible influence of granulometric composition on the mobility of metals due to high homogeneity [51]. The metal speciation analysis was carried out step by step according to the strict BCR procedure developed by the European Commission within the framework of the “Certified Reference Materials for the Quality Control of Chemical Speciation” project, improved by European researchers [29]. Reagents free of contaminants that could affect the analytical results (pure for analysis or chemically pure, Sigma Aldrich) were used. The validation of the BCR method involved various steps. To reduce errors, four determinations were made for each bentonite sample, and the reagent blanks were established. Coefficients of variation (CV) of <5% ensured the accuracy and reproducibility of the method. Recovery control was performed by comparing the total metal content (determined in a separate test) with the total metal content in all fractions. The efficiency was 95–105% and met the quality control standards. Metals were determined using inductively coupled plasma optical emission spectrometry (ICP-OES). Before the test, the instrument was calibrated using a multielement standard for ICP-OES (Instrument Calibration Standard 2, No. N9301721, PerkinElmer). At this stage, the linearity of the calibration curve, representing the relationship between the optical signal and the element concentration, was determined. The fit of the calibration curve was assessed using a linear regression statistical model with a significance level (*p =* 0.01). The background signal level was monitored during the experiments. The results were statistically analyzed to exclude gross errors using the Grubbs’ test.

### 2.3. Statistical Treatments

The mobility of potentially toxic metals in bentonites contaminated with Zn or Cu ions was analyzed using Statistica 12 software (StatSoft Inc., Tulsa, OK, USA), and the interpretation of the results was based on the guidelines provided by Rabiej [60]. Normality of the distribution was checked using the Kolmogorov–Smirnov test, while homogeneity of variance was assessed using Levene’s test. To assess how the independent variables, the origin of the clay and its contamination, affect the treated set of dependent variables, which were the fractions of potentially toxic metals (F_I_ + F_II_) Cu, Zn, Cr, Ni, and Pb, MANOVA analysis was used. In addition, interaction effects were tested between the bentonite origin and contamination to test more complex hypotheses. The discriminatory ability of the model was assessed using Wilks’ lambda values. Scheffe’s conservative post-hoc test was performed to determine the significance of differences between group averages of ‘origin of land’. Regression analysis was used to assess whether changes in the BCR Zn or Cu chemical fractions could be predicted from the independent variables (total Zn or Cu concentration and changes in unfrozen water content). The value of the coefficient of determination *R*^2^ describes the percentage of the linear model describing the variation in a specific metal chemical fraction (F_I_–F_IV_). The standard error of the estimate makes it possible to assess the deviation of the actual concentration of a given metal chemical fraction from the predicted value. The unstandardized coefficient (*B*) represents the values of the model coefficients. The standardized coefficient (*ß*) compares the strength of the effect of the independent variable on the dependent variable. The higher the absolute value of the beta coefficient, the stronger the effect. A test probability value of *p* < 0.05 allows the conclusion that the independent variables are statistically significantly related to the dependent variable.

## 3. Results and Discussion

### 3.1. The Properties of Bentonites

The physicochemical parameters of bentonites are summarized in Table 2. All potentially toxic elements are listed in Table A1 and Table A2 in the Appendix A.

### 3.2. Effect of Bentonite Origin and Main Exchangeable Cation Cu or Zn on Mobility of Potentially Toxic Metals

The percentages of individual BCR Cu or Zn fractions (F_I_—Exchangeable and weak acid soluble fractions; F_II_—Reducible fraction, F_III_—Oxidizable fraction, and F_IV_—Residual fraction) and the direction of their changes differed depending on the origin of the clay and the type of the main exchangeable cation. The proportion of the fraction of potentially mobile metals (F_I_ + F_II_) in each bentonite after contamination with Cu or Zn ions differed from those in the uncontaminated clays (Table A1 and Table A2). MANOVA showed a statistically significant effect of clay type, contamination with Zn or Cu ions, and the interaction of these factors on the entire set of potentially mobile metal fractions (F_I_ + F_II_) of Cu, Zn, Cr, Ni, and Pb (Table 3). Thus, the results do not exclude the possibility that the mobility of metals in model clays depends on both the origin of the clay and the type of the main exchangeable cation (Figure 1). Studies by Nartowska [24] and Nartowska et al. [57] confirmed the significant influence of the origin of the clay and the type of main exchangeable cation on shaping the physicochemical and structural properties of bentonites. Changes in these properties can result in different metal mobilities.

Significantly higher mobility of metals was observed in the Slovakian BSvk bentonite than in the American STx-1b and SWy-3 bentonites. It is most likely that the quality of bentonite is important here (Figure 1). For example, Dutta et al. [34] confirmed that bentonite quality affects its hydraulic properties. This, in turn, can be related to the transport of solutes into the clay pores. Scheffe’s post hoc test showed that the average content of potentially mobile metals varied depending on the origin of the bentonite, with the most significant differences occurring in BSvk bentonite contaminated with Cu ions (*p* = 0.001). Therefore, this clay was selected for further analysis. 

**Figure 3:**
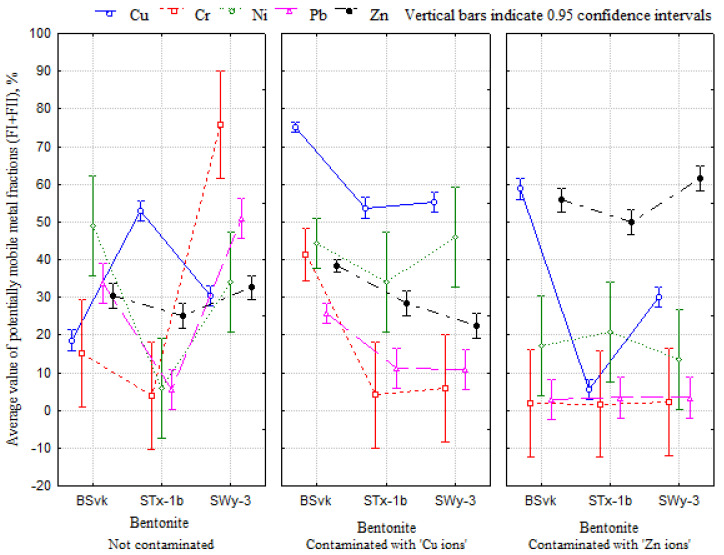
The average value of potentially mobile fractions (F_I_ + F_II_) of metals, depending on the type of bentonite and contamination. The results for BSvk “Cu’’ were shown independently of Cu concentration as the average of all samples tested.

**Figure 4:**
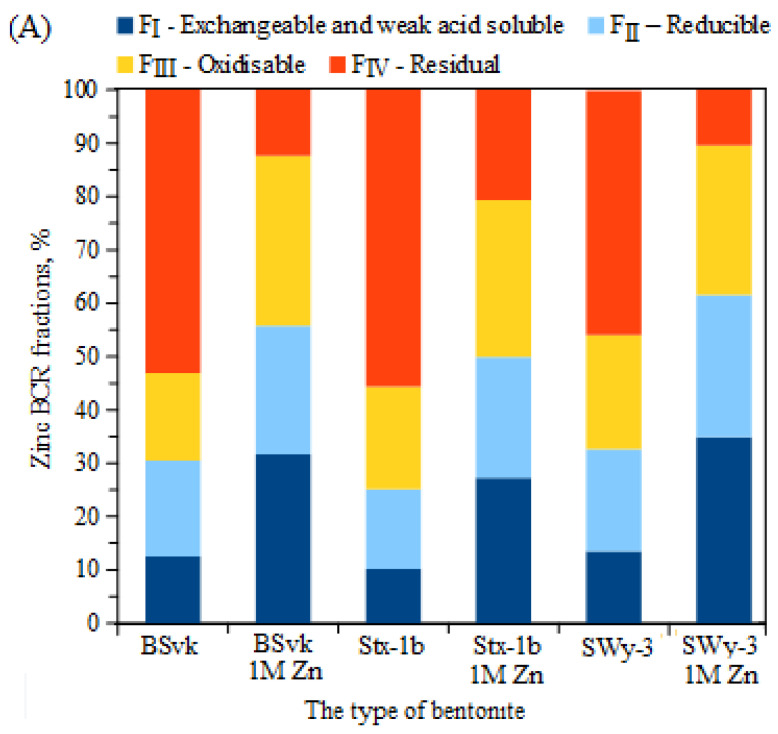

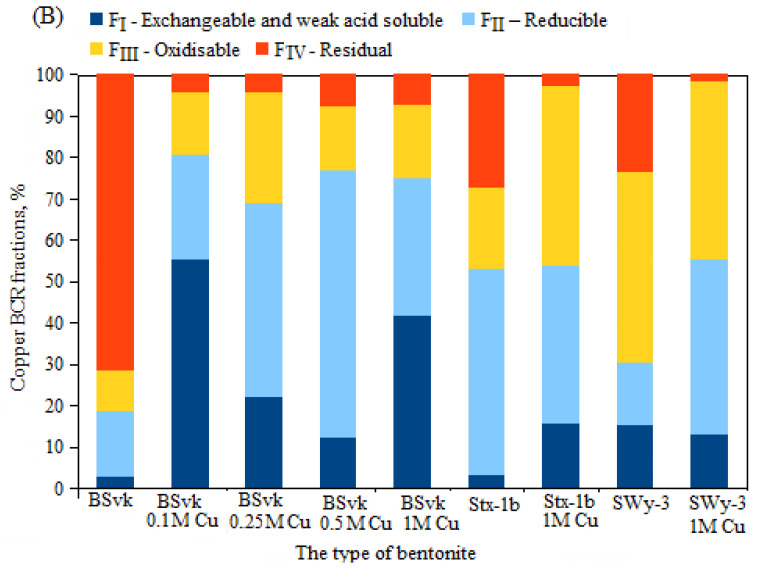
Contents of potentially toxic metals in BCR chemical fractions, depending on the type of bentonite and type of contamination: (**A**) clays contaminated with Zn ions and (**B**) clays contaminated with Cu ions.

The highest potential mobility of these metals was achieved in bentonites contaminated with Cu or Zn ions (Figure 3). Contamination of the clay with a particular metal increases mobility. Kashem et al. [31] confirmed that the anthropogenic origins of Cd, Zn, and Pb affect the increase in the proportion of their mobile fractions in contaminated soils in Japan. In the present study, a five-fold increase in the mobility rates of Cd and Zn and a two-fold increase in the mobility rate of Pb in clays contaminated with these metals were observed compared to uncontaminated clays. Similarly, Ma et al. [19] observed the highest concentrations of anthropogenic Cr and Cd in the mobile fraction. In this fraction, metals such as Pb, Co, Zn, and Ni were not observed, suggesting that these ions may form stable complexes with Fe and Mn oxides, and Co, Ni, and Cu may additionally be complex with humic substances. This phenomenon requires further investigation. Figure 4 shows that the mobility of metals in bentonites contaminated with Zn and Cu ions differs. The lowest mobilities of Pb and Ni were observed in the clays contaminated with Zn ions. Therefore, further analyses were conducted separately for clays contaminated with Cu and Zn ions.

### 3.3. The Effect of the Total Concentration of Zn or Cu on the Proportion of These Metals in Mobile and Non-Mobile Fractions

The proportion of mobile and stable fractions of Zn and Cu varied depending on the type of bentonite as well as the metal concentration (Figure 4). The Zn mobility in uncontaminated bentonites followed the pattern F_IV_ > F_II_ > F_III_ > F_I_ (BSvk) or F_IV_ > F_III_ > F_II_ > F_I_ (Stx-1b, SWy-3). The stable fraction dominated in all clays, confirming the suitability of bentonites for accumulating potentially toxic metals. These results are consistent with those reported in the literature [61,62]. After the bentonite was contaminated with Zn ions, the proportion of individual chemical fractions changed significantly. The dominant directions were F_III_ > F_I_ > F_II_ > F_IV_ (BSvk, Stx-1b) or F_I_ > F_III_ > F_II_ > F_IV_. In any bentonite contaminated with Zn ions, the smallest share was the immobile fraction, F_IV_, and the mobile fraction, F_I_, had a high share. The smallest proportion of non-mobile fractions after Zn ion contamination was observed in Wyoming bentonite, originally sodium bentonite. Copper mobility in uncontaminated bentonites followed the pattern F_IV_ > F_II_ > F_III_ > F_I_ and for samples contaminated with Cu ions, F_I_ > F_II_ > F_III_ > F_IV_ or F_II_ > F_III_ > F_I_ > F_IV_. In each case, the proportion of stable fractions decreased significantly after the samples were contaminated with Cu ions. Similarly, Liu et al. [63] observed that the availability of Cu and Cd was negatively correlated with the percentage of the residual fraction of metals.

The results of the regression analysis confirmed a statistically significant relationship between the total Zn ion content in the clay and its content in the individual Zn fractions determined with the BCR method (F_I_–F_IV_) (Table 4).

An increase in Zn concentration had a statistically significant (*p* < 0.05) effect on the increase in mobile fractions (F_I_, F_II_) and a decrease in stable fractions (F_III_, F_IV_), regardless of the origin of the bentonite. In view of the marked disparity in the content of mobile Cu fractions in bentonites of different origins (Figure 4B) and the significant Scheffe test, a study of the effect of Cu ion concentration on the proportion of copper BCR fractions was carried out on BSvk bentonite. Statistically significant relationships were obtained only for the mobile fractions (F_I_ and F_II_). With an increase in the concentration of Cu ions, the proportion of fraction I decreased, indicating a different behavior from that of bentonites contaminated with Zn ions. Most likely, in clays contaminated with Cu ions, the higher the concentration of Cu, the greater the proportion of ions trapped in the structure of bentonites, while simultaneously reducing their mobility under exchangeable fraction conditions. This was confirmed in an earlier study by Nartowska and Kozłowski [47], which showed that, in bentonites contaminated with Cu ions, the presence of a new mineral phase, atacamite, increased in proportion with increasing Cu ion concentration. The possible formation of metal compounds, resulting in a reduction in metal mobility, was also reported by Zhang et al. [64]. According to the authors, higher soil pH may promote the adsorption of positively charged heavy metal ions by soil colloids and may increase ion binding with OH^−^ groups.

In addition to assessing the mobility of the main exchangeable ions Zn and Cu, certain dependencies in the mobility of co-occurring ions in the bentonite were observed. In both Zn- and Cu-ion-contaminated bentonites, relationships were observed between the concentration of metals co-occurring in these clays, such as Cu, Cr, Ni, and Pb, or Cr, Ni, Pb, and Zn, and their mobility. Moreover, their behavior in both clays was the opposite. Similarly, Nartowska [24] showed different behaviors of bentonites contaminated with Zn and Cu ions. Wang et al. [65] confirmed that the presence of co-occurring ions in the structure of bentonites is one of the factors, in addition to specific surface area, pore size, organic matter content, pH, and temperature, that affect the rate of ion release from the clay structure, in this case, phosphates.

In the case of bentonites contaminated with Zn ions, the following statistically significant relationships (*p* < 0.05) were confirmed: an increase in Cu concentration with an increase in Cu mobility index, an increase in Cr concentration with an increase in Cr mobility index and the content of potentially mobile Cr fractions (F_I_ + F_II_), an increase in Ni and Pb concentration affected the decrease in potentially mobile fractions of these metals (F_I_ + F_II_), and an increase in Cu, Cr, and Ni concentration affected the increase in the Pb mobility index.

In the case of bentonites contaminated with Cu ions, statistically significant relationships (*p* < 0.05) were confirmed: an increase in Cr content decreased the mobility index, and an increase in Ni content increased it. An increase in Pb content is related to an increase in the potentially mobile fraction (F_I_ + F_II_) of this metal. The total concentrations of Cr, Ni, and Zn affected the decrease in the mobility of Pb. The observed correlations, as they involve ions co-occurring in the soil structure with theoretically low concentrations of these metals, they do not pose a threat to the environment; but may provide a basis for further research using bentonites in which Cr, Ni, and Pb ions predominate.

### 3.4. The Relationship between the Physicochemical Properties of Bentonites and the Mobility of Potentially Toxic Metals

Using regression analysis, it was assessed whether changes in physicochemical properties of bentonites, such as clay and silt fraction content, effective diameter (d_10_), Sauter mean diameter (d_32_), and interplane distance d_001_, are correlated with the content of individual metals (Zn, Cu, Cr, Ni, and Pb) in potentially mobile (F_I_ + F_II_) and mobile (F_I_) fractions.

The analysis showed statistically significant relationships (*p* < 0.05) in bentonites contaminated with Zn ions between the content of potentially mobile Cr, Zn, and Ni fractions and the clay fraction content (R_Cr,Zn_ = 0.99, R_Ni_ = −0.99). The content of potentially mobile Cu fractions increased with an increasing silt fraction. The content of potentially mobile Pb fractions increased with a decrease in the specific surface area of the clay and a decrease in the silt fraction. Therefore, Pb may accumulate in coarser fractions. The higher the clay fraction content, the lower the proportion of mobile fractions (F_I_) of Cu, Cr, Pb, and Zn. For Ni, the proportion of mobile fractions increased with increasing Ni concentration. Metal mobility (F_I_) is also strongly influenced by the granulometric parameters d_10_ and d_32_. The proportion of mobile fractions of Cu, Cr, Pb, and Zn increased with an increase in these parameters and a decrease in Ni. One could assume that the leachability of these metals is related to the ions trapped in the clay pores. Generally, the larger the effective diameters, the larger the pores. The higher mobility of Cu, Cr, Pb, and Zn metals with a decrease in d_001_ may suggest that ions located in larger pores are released at the F_I_ stage. This occurs because a decrease in d_001_ leads to a decrease in microporosity [57]. Conversely, this is the case for Ni. Similarly, Angelaki et al. [66] observed an increase in filtration rate, particle aggregation, and porosity in clay soils contaminated with Zn or Cu ions.

The relationship between the Cu mobile fraction (F_I_) and clay fraction, d_32_, and interpacket distance was significant, but in the opposite direction from that of bentonites contaminated with Zn ions. Changes in the behavior of metals that co-occurred in the clay structure (Cr, Ni, and Pb) were also observed. The proportion of the Cr mobile fraction (F_I_) increased with decreasing specific surface area and d_001_. The stability index of Ni (Ir) increased with an increase in the specific diameter and a decrease in the d_001_. The Pb mobility index increased with increasing effective diameter and decreasing d_001_, specific surface area, and clay fraction content.

Due to the different behaviors of metals in bentonites contaminated with Zn or Cu ions, it seems reasonable to learn about the influence of individual metals in bentonites where they occur as the main exchangeable cation. The data presented herein can provide a basis for further analysis.

#### The Impact of the Predominant Exchangeable Cation in Bentonites, Whether Zinc or Copper

Because the physicochemical properties of bentonites are determined by the type of main exchangeable cation [57], the most significant relationships between physicochemical parameters and the mobility of potentially toxic metals can be expected by evaluating the influence of Zn or Cu ions.

**Figure 5:**
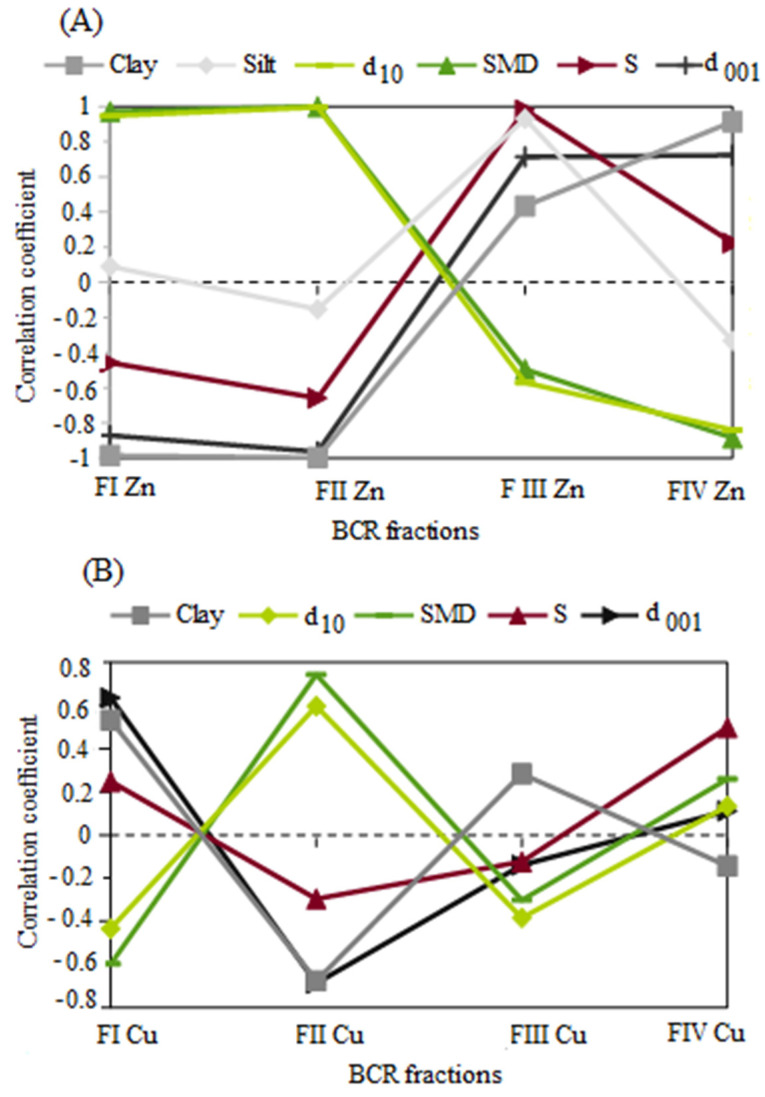
Correlation coefficients of clay parameters with individual chemical fractions of BCR metals (F_I_–F_IV_). (**A**) Zinc fractions in bentonites contaminated with zinc ions. (**B**) Copper fractions in bentonites contaminated with copper ions. Bentonite parameters tested: Clay—d < 0.002 mm, Silt—d (0.002–0.063 mm), d_10_—effective diameter is that size below which 10% of the material is contained, SMD—Sauter Mean Diameter is also referred to as d_32_ (Laser diffraction method); d_001_—interpacket distance (XRD method); S—specific surface (Water sorption test method).

It was established that the mobility of Zn or Cu is related to the changes in the physicochemical properties of the bentonites resulting from their introduction into the structure of bentonites due to ion exchange (Figure 5). Statistically significant correlations (*p* < 0.05) were established in bentonites contaminated with Zn ions (Figure 5A) between F_IZn_ and F_IIZn_ and clay fraction content, d_001_, d_10_, and d_32_. Significant (*p* < 0.05) correlations in bentonites contaminated with Cu ions (Figure 5B) were obtained for F_ICu_ and F_IICu_ with clay fraction content, d_001_ and d_32_, and for F_IICu_ additionally with d_10_. For F_IVCu_ with specific surface area, the correlations were not significant.

**Figure 6:**
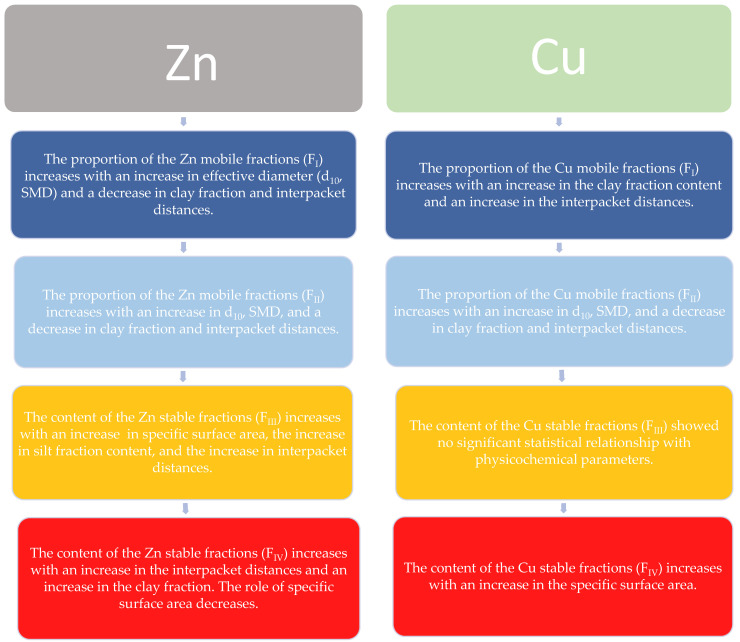
The relationship between the content of BCR chemical fractions and the physicochemical parameters of bentonites, depending on the type of main exchange cation, Zn or Cu.

Figure 6 shows the relationship between the different chemical fractions and physicochemical properties, depending on the type of metal, Zn or Cu. The results indicate different behavior of these bentonites and clarify the results shown in Figure 5. Different colors indicate the four successive stages of the BCR sequential extraction. Dark blue represents stage 1, while red represents stage 4.

Individual stages in the BCR method correspond to metal ions present in different chemical forms in the soil–water environment: Stage 1, metals in exchangeable form or bound to carbonates (F_I_); Stage 2, metals bound to amorphous iron and manganese oxides (F_II_); Stage 3, metals bound to organic matter or sulfides (F_III_); and Stage 4, metals bound to primary and secondary minerals (F_IV_). Metals released in stages 1 and 2 are considered to pose a threat to the environment due to their high mobility, whereas metals released in stages 3 and 4 are considered to have no significant impact on the environment due to their high stability.

Based on the correlation coefficients assessment (Figure 5) and the diagram (Figure 6), the likely mechanism of Zn or Cu mobility in bentonites depends on clay properties, and the type of chemical fraction determined via the BCR method was established.

For Zn bentonites, it was observed that in stage 1 of the BCR, Zn ions accumulate in larger pores where free water is present. These observations are confirmed by the decrease in interpacket distances, which is closely related to the decrease in microporosity [57]. In turn, a decrease in microporosity indicates an increase in the proportion of pores with larger diameters. At stage 2 of the BCR method, higher clay fraction contents and shorter d_001_ distances resulted in reduced mobility of Zn ions. This suggests that microporosity may have further decreased, as indicated by the lower correlation coefficient for d_001_ compared to F_I_. The increase in Zn ions with increasing effective diameters indicates that Zn is still released from free water located in larger pores. At stage 3, it was observed that the stability of Zn ions increases with increasing specific surface area, increasing silt fraction content, and increasing interpacket distances. Most likely, Zn accumulates in the interpacket space and on the mineral surface. The association with the silt fraction may result from particle aggregation after contamination with Zn ions. Zn ions are likely to be present in bound water (interlayer water and diffuse double-layer water). At stage 4, the stability of Zn ions increases with increasing clay fraction content and increasing interpacket distances. Larger interpacket distances probably lead to a higher proportion of micropores [57]. Zn ions accumulating in micropores are bound to bentonite particles and may be difficult to access for ion exchange.

For Cu bentonites, at stage 1 of the BCR, it was observed that the proportion of the Cu mobile fraction increases with the increase in clay fraction content and interpacket distances. Cu ions are likely released from the interpacket spaces of the bentonite. The larger the space between packets, the more Cu ions can be contained and released more easily. The highest clay fraction content and the largest interpacket distances were observed in the bentonite ‘BSvk 1M Cu’. Lower values were found in the bentonites ‘BSvk 0.1–0.5 M Cu’. It can be assumed that the high proportion of the mobile copper fraction is associated with the mineralogical composition of the bentonites. Bentonites ‘BSvk 0.1–0.5 M Cu’ contain a few percent of copper-binding minerals (atacamites) in their structure [47]; hence, in such bentonites, the mobility of Cu may be impended. At stage 2 of the BCR, Cu ions are released from water located in larger pores of the bentonite, as evidenced by the increase in effective diameters with the increase in the amount of released ions. Stage 3 of the BCR did not show statistically significant relationships between the proportion of potentially stable Cu fractions and the physicochemical parameters of the bentonite. The content of the stable Cu fraction at stage 4 of the BCR increased with the increase in the specific surface area of the bentonites. Conversely, the specific surface area increased with the increase in the ionic strength of the copper chloride (II) solution used for ion exchange (0.1M–1M). It is likely that a large portion of Cu ions is located on the surface of bentonite particles and is bound to them by attractive forces, which increase with higher molar concentrations of the solution. Hao et al. [43] confirmed that at low ionic strength of the solution, the removal capacity of Cu from sodium bentonite is the highest.

The results obtained were partially consistent with those reported by Ma et al. [19]. The authors studied river sediments from mining areas in China and concluded that the Cu, Zn, Cd, and As residual fractions dominated in sediments with grain sizes larger than 150 μm, while the other three BCR fractions inversely dominated in fractions smaller than 150 μm. Although the research carried out in this article deals with bentonites with grain sizes < 63 μm, it can be observed that the contribution of fractions I-III of Zn or Cu is the largest and the residual fraction is the smallest. However, the results of this study suggest that the relationships between granulometric composition and BCR chemical fractions are more complex, as described above, and depend strongly on the type of Zn or Cu metal and the type of BCR fraction. A different mechanism of Zn or Cu mobility was also observed by Alloway et al. [67], who noted that the mobility of Zn is higher than that of Cu because Cu is strongly retained by the soil solid phase. Similarly, a strong association between Cu ions and the specific surface of the soil is evident in the presented results.

### 3.5. Mobility of Zn or Cu at Freezing Temperatures

**Figure 7:**
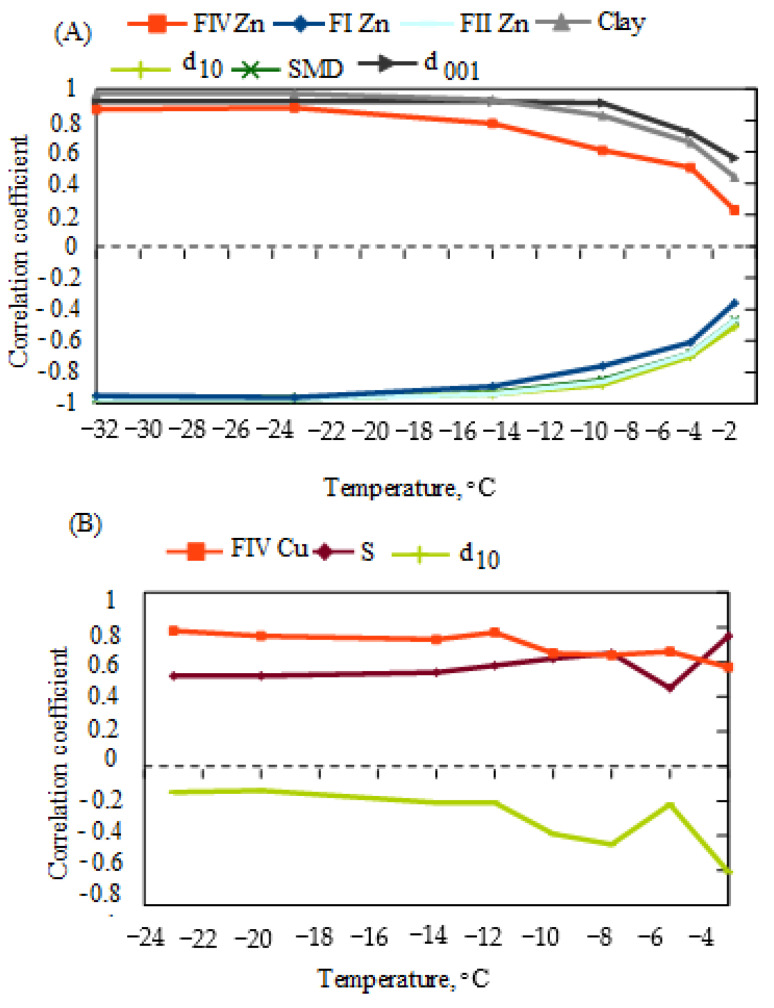
The best correlations of unfrozen water content with metal chemical fractions and clay properties (**A**) in bentonites contaminated with Zn ions and (**B**) in bentonites contaminated with Cu ions. Clay studied properties: Clay—d < 0.002 mm, d_10_—effective diameter is that size below which 10% of the material is contained, SMD—Sauter Mean Diameter is also referred to as d_32_ (Laser diffraction method); d_001_—interpacket distance (XRD method); and S—specific surface area (Water sorption test method). The unfrozen water content was taken from the authors’ published results for the same bentonite samples (Nartowska et al. [26], Figures 2–4; Nartowska and Kozłowski [47], Table 6). The unfrozen water content was determined using the method of 1HNMR (bentonites contaminated with Zn ions) in the following temperatures: −32 °C, −23 °C, −14 °C, −9 °C, −5 °C, and −3 °C and by using differential scanning calorimetry, DSC, with the following temperatures: −23 °C, −20 °C, −14 °C, −12 °C, −10 °C, −8 °C, −6 °C, −4 °C, and −2 °C.

Bentonites subjected to Zn or Cu ion contamination, as well as additionally exposed to freezing–thawing cycles, constitute a complex clay–water system. Barker et al. [68] suggest that the mobilization of trace metals is seasonally dependent. At subzero temperatures, there exists a fraction of liquid water acting as a carrier for potentially toxic metals, and its quantity may be associated with the interaction of the ions themselves and changes in the properties of bentonites due to their introduction to the structure of bentonite [25,26]. Rui et al. [69], on the other hand, argue that freezing–thawing can lead to the disaggregation of soil particles, resulting in a higher proportion of Cd and Pb metals in the thawing front, although they emphasize the significance of soil particle size and specific surface area. Consequently, the correlational relationships between the unfrozen water content at specific negative temperatures with BCR Zn or Cu chemical fractions and clay properties were assessed (Figure 7).

Additionally, to statistically confirm significant relationships between individual BCR Zn or Cu fractions for bentonites contaminated with Zn or Cu ions and unfrozen water content at specific negative temperatures, regression analysis was conducted (Table 5).

The results of changes in unfrozen water content at selected temperatures are presented in Table A3.

Figure 7 shows the best correlations of unfrozen water content at a given freezing temperature with chemical fractions determined using the BCR method: F_IZn_, F_IIZn_, F_IVZn_, and F_IVCu_, clay, S, d_001_, d_10_, and d_32_.

The proportion of exchangeable and reducible fractions of Zn (F_IZn_ and F_IIZn_) correlated significantly and in the same direction as the d_10_ and d_32_ in bentonites contaminated with Zn ions (Figure 7A). An increase in unfrozen water content was observed with a decrease in F_IZn_ content, F_IIZn_ content at a given negative temperature, effective diameter, and Sauter diameter. The decrease in Zn concentration in the fractions (F_IZn_ and F_IIZn_) of Zn bentonites is probably a consequence of a decrease in the d_10_ due to contamination of bentonites with Zn ions. The increase in microporosity causes some Zn ions to be trapped in small pores, making them more difficult to elute.

The content of Cu stable fractions (F_IV_) in the Cu bentonites increased with increasing unfrozen water content and specific surface area at a given negative temperature (Figure 7B). The trend of changes in correlation coefficients with unfrozen water content for F_IV_ and S was similar at temperatures ≤ −12 °C. It can be assumed that the specific surface area of the clay had a significant effect on the changes in the stable fractions in the given temperature range. The effect of specific surface area on the proportion of Cu stable fractions (F_IVCu_) seems less significant at temperatures ≥ −6 °C. This may be because, near 0 °C, unfrozen water is mainly water trapped in mesopores whose degree of filling is less related to the specific surface area of the bentonite. An antagonistic trend was observed in the relationship between the correlation coefficients of unfrozen water content with specific surface area and the correlation coefficients of unfrozen water with effective diameter at individual negative temperatures (Figure 7B).

As the relationship between unfrozen water content and Zn content in the F_IZn_ mobile fraction is statistically significant at temperatures ≤ −14 °C (Table 5), it can be assumed that the relationships observed in Figure 4A for F_IZn_ and d_10_ are most significant in this temperature range. Most likely, the decrease in total zinc concentration, which is statistically significant in this temperature range, leads to a decrease in the proportion of F_IZn_ and an increase in unfrozen water content. In turn, the proportion of Zn in the F_I_ mobile fraction decreases with a decrease in the effective diameter, which can be explained by the fact that the volume of connected pores filled with water decreases owing to thawing [70]. Thus, Zn ions trapped in the smaller pores become more difficult to leach. At the same negative temperatures, the increase in the proportion of Zn stable fractions with increasing unfrozen water content can also be explained by a decrease in pore size and by trapping some Zn ions in the bentonite interpacket space. This was evidenced by the significant correlation between F_IVZn_ and d_001_. The phenomenon where a lower proportion of mobile Zn fractions occurs at higher unfrozen water contents does not imply the absence of risk to the groundwater environment under negative temperature conditions. Higher levels of unfrozen water are observed in bentonites with lower total Zn ion concentrations. This means that the proportion of mobile Zn fractions is lower in bentonites with lower total Zn concentrations.

In bentonites contaminated with Cu ions, there was a statistically significant relationship (*p* < 0.05) between the increase in unfrozen water content at individual negative temperatures and the increase in the proportion of stable Cu fractions (F_IVCu_) (Table 5). At temperatures ≤ −12 °C, these changes were consistent with changes in specific surface area. The content of unfrozen water at a given negative temperature is higher when the total concentration of Cu ions in the clay is low [47]. Thus, higher contamination of bentonites with Cu ions leads to a decrease in the proportion of Cu-stable fractions with a decrease in the amount of unfrozen water. These results confirm previous findings [24] that granulometric parameters are more responsible for the behavior of bentonites contaminated with Zn ions, and that the behavior of bentonites contaminated with Cu ions is influenced by specific surface area parameters. It seems that, at subzero temperatures, relationships with the same clay properties are important. The results presented suggest, under freezing temperature conditions, the presence of concentrated Zn or Cu solutions in bentonites. A study by Wu et al. [71] confirmed that cyclic freezing–thawing enhanced the contact reaction between the solid phase and the liquid, leading to an increase in the removal efficiency of Cd and Pb metals from the front of the freeze–thaw and thus the formation of concentrated solutions. On the other hand, experiments were carried out with the addition of an eluent, EDTA with tartaric acid or citric acid, whose contact with heavy metals in low-permeability clay soils can be limited, which can reduce the effectiveness of remediation.

The main limitations of this study concern the BCR procedure, which is labor-intensive and sensitive to slight changes in the procedure. The extracted fractions, defined operationally, roughly characterize the forms of metal occurrence in the clay. In addition, there is a lack of standardized sample preparation procedures for testing. Despite its many drawbacks, the sequential extraction method is an important source of information on the bioavailability and stability of potentially toxic metal ions in the environment [2].

## 4. Conclusions

Based on the analysis of the results, the following conclusions were drawn:

1. The mobility of potentially toxic metals such as Cu, Zn, Cr, Ni, and Pb in bentonites is primarily determined by the type of main exchangeable cation, whether Zn or Cu, its total concentration, and, to a lesser extent, the origin of the bentonite. Further studies on bentonites containing Cr, Ni, and Pb as the main exchange cations are necessary to assess their mobility;
2. High total concentrations of Zn ions in Zn bentonites led to increased mobility of Zn ions while simultaneously decreasing the proportion of stable fractions. Conversely, in Cu bentonites, increasing Cu concentrations resulted in decreased Cu mobility in stage 1 of the BCR method, likely due to the binding of Cu ions to the mineral phase of atacamite;
3. There is a statistically significant relationship (*p* < 0.05) between Zn or Cu mobility and the structural and physicochemical properties of bentonites. The proportion of Zn mobile fractions (F_IZn_) increased with increasing effective diameter, decreasing clay fraction content, and interpacket distances, while the proportion of Cu mobile fractions (F_ICu_) increased with decreasing effective diameter and increasing clay fraction content and interpacket distances. Zn ions likely accumulate in larger soil pores, while Cu ions are immobilized in the sorption complex of bentonite;
4. The stability of potentially toxic Zn or Cu metals in bentonites increases with increasing clay fraction content or, respectively, with increasing specific surface area;
5. It is possible that, at subzero temperatures, unfrozen water forms a concentrated solution of Zn or Cu, which may pose a threat to the groundwater environment, especially in cold regions.

## Figures and Tables

**Table 1:** The BCR procedure [52].

Step No.	Procedure/Condition	Fraction
1	Weigh 2 g of dry mass of each bentonite and transfer to a 100 cm^3^ rotary tube. Add 40 cm^3^ of 0.11 mol/dm^3^ CH_3_COOH. Shake for 16 h (T = 22 °C). Separate extract via centrifugation.	**F_I_**—Exchangeable and/or carbonate-bound metals
2	Flood clay samples with 40 cm^3^ of 0.5 mol/dm^3^ NH_2_OH∙HCl, pH = 2. Use HNO_3_ to correct reaction. Perform procedure as in step one.	**F_II_**—Metals combined with amorphous Fe and Mn oxides
3	Add 10 cm^3^ of 8.8 mol/dm^3^ H_2_O_2_ to remaining clay samples after step 2. Place in water bath at 85 °C until solution evaporates. Pour cooled samples into 50 cm^3^ ammonium acetate solution (1 mol/dm^3^, pH = 2 after HNO_3_ correction). Shake for 16 h and centrifuge to separate clay precipitate from extract.	**F_III_**—Metals bound to organic matter and sulfides
4	Dry remaining sediment at 105–110 °C. Place 1 g of clay in a 300 cm^3^ conical volumetric flask. Add 7.5 cm^3^ concentrated HCl and 2.5 cm^3^ concentrated HNO_3_. Heat until the solution evaporates. Add 25 cm^3^ of HCl (1 + 5) to the cooled flasks, transfer the dissolved precipitate to a volumetric flask and fill up to 50 cm^3^ with distilled water. Filter the solution and determine the metals in fraction IV.	**F_IV_**—Metals bound to primary and secondary minerals

**Table 2:** Properties of the bentonites [26,47].

Properties	BSvk	BSvk Cu				BSvk 1 M Zn	Stx-1b	Stx-1b 1M Cu	Stx-1b 1 M Zn	SWy-3	SWy-3 1 M Cu	SWy-3 1 M Zn
		1 M	0.1 M	0.25 M	0.5 M							
Cu ^&^ (mg/kg)	6.28 ± 0.06	7677 ± 70	23,284 ± 121	27,571 ± 90	28,773 ± 162	39.55 ± 0.25	8.97 ± 0.13	5427 ± 43	52.30 ± 0.53	12.80 ± 0.15	11,221 ± 61	109.47 ± 1.34
Zn ^&^ (mg/kg)	64.54 ± 0.69	95.61 ± 0.72	71.36 ± 2.13	78.72 ± 0.86	80.75 ± 2.03	17,857 ± 89	73.68 ± 0.27	92.96 ± 2.91	16,153 ± 75	163.66 ± 1.50	83.20 ± 1.30	44,463 ± 124
Na ^&^ (mg/kg)	1151 ± 9	413 ± 5	168 ± 1	195 ± 2	133 ± 2	1204 ± 22	1970 ± 18	363 ± 7	885 ± 16	10,086 ± 81	405 ± 6	995 ± 9
Ca ^&^ (mg/kg)	11,945 ± 140	1598 ± 28	676 ± 23	328 ± 6	385 ± 5	2778 ± 31	11,802 ± 101	1491 ± 19	2985 ± 23	8282 ± 57	2028 ± 21	4526 ± 34
clay * (%)	19.8	20.5	18	18.5	16	8.9	18.5	23.5	10.7	42.6	13.2	6.55
silt * (%)	80.2	79.5	82	81.5	84	90.2	81.5	76.5	87	57.4	86.8	87
d_10_ * (μm)	1.33	1.31	1.44	1.39	1.52	2.18	1.39	1.26	1.91	0.96	1.71	2.83
d_32_ * (μm)	3.16	3.01	3.26	3.22	3.56	5.22	3.44	2.85	4.57	1.88	4.07	6.31
S ^#^ (m^2^/g)	671	460	183	189	203	557	568	414	538	307	355	516
d_001_ ^+^ (Å)	14.88	12.48	12.38	12.34	12.31	14.17	14.88	12.48	14.29	11.44	12.32	13.21

^&^ Total content of major elements in the dry clay matrix determined with ICP-OES. All potentially toxic elements are contained in Table A1 and Table A2; * clay (d ≤ 0.002 mm), silt (0.002 mm < d < 0.063 mm), d_10_ (arithmetic mean diameter), and d_32_ (Sauter mean diameter) determined with Laser diffraction method; ^#^ Specific surface area determined with Water sorption test method; ^+^ interpacket distance determined with XRD method; ± Standard deviation.

**Table 3:** Multivariate tests of the significance of clay, contamination, and their interactions for the potentially mobile metal fractions (F_I_ + F_II_) of Cu, Zn, Cr, Ni, and Pb.

Parameter	Wilks—Test Value	F-Test Value	Degrees of Freedom (df)	df Error	*p*-Value	Significance
Potentially mobile fraction (F_I_ + F_II_) of Cu, Zn, Cr, Ni, Pb						
Intercept	0.003	3796.845	3	37	0.000	
Bentonite (B)	0.084	30.143	6	74	0.000	***
Contamination (C)	0.017	82.705	6	74	0.000	***
B × C	0.007	45.217	12	98.184	0.000	***

Bentonite: BSvk, STx-1b, SWy-3; Contamination: Clay non-contaminated. Bentonites contaminated with ‘Cu ions’, Bentonites contaminated with ‘Zn ions’; *** Significant at the 0.001 probability level.

**Table 4:** Regression analysis between the total concentration of Zn^2+^ ions and the amount of Zn^2+^ ions in each BCR fraction (F_I_–F_IV_).

	The Standardized Beta (ß)	Std. Error ß	The Unstandardized Beta (B)	Std. Error B	*t* Test Value	*p*-Value	Significance
**Dependent variable: F_I_ Zn/Cu**							
**R = 0.847 R^2^ = 0.717 adj. R^2^ = 0.689 Std. error of estimate: 1.81**							
**Intercept**			25.976	1.180	22.011	0.000	
**Zn total**	0.847	0.168	0.000	0.000	5.035	0.000	***
**R = 0.522 R^2^ = 0.273 adj. R^2^ = 0.221 Std. error of estimate: 15.26**							
**Intercept**			55.465	10.603	5.231	0.000	
**Cu total**	−0.522	0.228	−0.001	0.000	−2.291	0.038	*
**Dependent variable: F_II_ Zn/Cu**							
**R = 0.950 R^2^ = 0.903 adj. R^2^ = 0.894 Std. error of estimate: 0.58**							
**Intercept**			21.218	0.375	56.561	0.000	
**Zn total**	0.950	0.098	0.000	0.000	9.664	0.000	***
**R = 0.562 R^2^ = 0.316 adj. R^2^ = 0.267 Std. error of estimate: 13.09**							
**Intercept**			20.880	9.092	2.296	0.038	
**Cu total**	0.562	0.221	0.000	0.000	2.543	0.023	*
**Dependent variable: F_III_ Zn/Cu**							
**R = 0.746 R^2^ = 0.557 adj. R^2^ = 0.513 Std. error of estimate: 1.14**							
**Intercept**			32.097	0.744	43.162	0.000	
**Zn total**	−0.746	0.210	−0.000	0.000	−3.547	0.005	**
**R = 0.185 R^2^ = 0.034 adj. R^2^ = 0.004 Std. error of estimate: 4.92**							
**Intercept**			16.549	3.417	4.842	0.000	
**Cu total**	0.185	0.262	0.000	0.000	0.706	0.492	NS
**Dependent variable: F_IV_ Zn/Cu**							
**R = 0.690 R^2^ = 0.476 adj. R^2^ = 0.424 Std. error of estimate: 3.54**							
**Intercept**			20.709	2.299	9.008	0.000	
**Zn total**	−0.690	0.229	−0.000	0.000	−3.014	0.013	**
**R = 0.275 R^2^ = 0.075 adj. R^2^ = 0.009 Std. error of estimate: 1.668**							
**Intercept**			7.106	1.158	6.134	0.000	
**Cu total**	−0.275	0.257	−0.000	0.000	−1.070	0.303	NS

ƩN = 112 Significant at the *** 0.001; ** 0.01; * 0.05 probability level; NS—no significant.

**Table 5:** Regression analysis between the unfrozen water content in a given temperature and the amount of Zn^2+^ and Cu^2+^ ions in a given BCR fraction (F_I_-F_II_-F_IV_).

	The Standardized Beta (ß)	Std. Error ß	The Unstandardized Beta (B)	Std. Error B	*t*-Test Value	*p*-Value	Significance
Dependent variable: **F_I_ Zn**							
R = 0.949 R^2^ = 0.902 adj. R^2^ = 0.888 Std. error of estimate: 0.819							
Intercept			34.592	2.755	12.555	0.000	
Un ^$^ (T = −32 °C)	−0.949	0.118	−0.702	0.087	−8.011	0.000	***
R = 0.956 R^2^ = 0.915 adj. R^2^ = 0.903 Std. error of estimate: 1.031							
Intercept			38.767	2.775	13.969	0.000	
Un ^$^ (T = −23 °C)	−0.956	0.110	−0.765	0.088	−8.673	0.000	***
R = 0.887 R^2^ = 0.787 adj. R^2^ = 0.757 Std. error of estimate: 1.352							
Intercept			40.960	4.551	8.999	0.000	
Un ^$^ (T = −14 °C)	−0.887	0.174	−0.736	0.145	−5.089	0.001	***
Dependent variable: **F_II_ Zn**							
R = 0.968 R^2^ = 0.937 adj. R^2^ = 0.928 Std. error of estimate: 0.656							
Intercept			44.820	3.166	14.156	0.000	
Un ^$^ (T = −32 °C)	−0.968	0.095	−1.316	0.129	−10.193	0.000	***
R = 0.972 R^2^ = 0.946 adj. R^2^ = 0.938 Std. error of estimate: 0.659							
Intercept			49.829	3.180	15.669	0.000	
Un ^$^ (T = −23 °C)	−0.972	0.088	−1.432	0.129	−11.036	0.000	***
R = 0.936 R^2^ = 0.876 adj. R^2^ = 0.858 Std. error of estimate: 1.034							
Intercept			52.863	4.992	10.590	0.000	
Un ^$^ (T = −14 °C)	−0.936	0.133	−1.429	0.203	−7.019	0.000	***
R = 0.857 R^2^ = 0.734 adj. R^2^ = 0.668 Std. error of estimate: 2.196							
Intercept			65.323	12.984	5.031	0.007	
Un ^$^ (T = −9 °C)	−0.857	0.257	−1.763	0.529	−3.329	0.029	*
R = 0.683 R^2^ = 0.466 adj. R^2^ = 0.390 Std. error of estimate: 1.806							
Intercept			48.418	8.720	5.552	0.001	
Un ^$^ (T = −5 °C)	−0.683	0.276	−0.879	0.355	−2.474	0.042	*
Dependent variable: **F_IV_ Zn**							
R = 0.873 R^2^ = 0.762 adj. R^2^ = 0.728 Std. error of estimate: 1.273							
Intercept			6.091	1.443	4.218	0.004	
Un ^$^ (T = −32 °C)	0.873	0.184	0.450	0.095	4.736	0.002	**
R = 0.882 R^2^ = 0.778 adj. R^2^ = 0.746 Std. error of estimate: 1.332							
Intercept			7.668	1.510	5.076	0.001	
Un ^$^ (T = −23 °C)	0.882	0.178	0.493	0.099	4.951	0.002	**
R = 0.784 R^2^ = 0.615 adj. R^2^ =0.559 Std. error of estimate: 1.820							
Intercept			11.316	2.064	5.482	0.000	
Un ^$^ (T = −14 °C)	0.784	0.235	0.455	0.136	3.341	0.012	*
Dependent variable: **F_IV_ Cu**							
R = 0.895 R^2^ = 0.802 adj. R^2^ = 0.782 Std. error of estimate: 1.024							
Intercept			13.850	1.123	12.328	0.000	
Un ^&^ (T = −23 °C)	0.895	0.141	1.159	0.182	6.364	0.000	***
R = 0.899 R^2^ = 0.807 adj. R^2^ = 0.788 Std. error of estimate: 0.892							
Intercept			16.675	0.979	17.037	0.000	
Un ^&^ (T = −14 °C)	0.899	0.139	1.028	0.159	6.480	0.000	***
R = 0.848 R^2^ = 0.720 adj. R^2^ = 0.692 Std. error of estimate: 0.858							
Intercept			20.980	0.941	22.290	0.000	
Un ^&^ (T = −10 °C)	0.848	0.167	0.773	0.153	5.069	0.000	***
R = 0.599 R^2^ = 0.359 adj. R^2^ = 0.295 Std. error of estimate: 2.622							
Intercept			26.778	2.877	9.308	0.000	
Un ^&^ (T = −4 °C)	0.599	0.253	1.105	0.466	2.369	0.039	*

ƩN = 147, Significant at the *** 0.001; ** 0.01; * 0.05 probability level; ^$^ Unfrozen water content in given temperatures determined using ^1^ H NMR method based on Nartowska et al. [26] ^&^ Unfrozen water content in given temperatures determined using DSC method based on Nartowska and Kozłowski [32].

## Data Availability

The original contributions presented in this study are included in the article; further inquiries can be directed to the corresponding author.

## Appendix A

**Table A1:** Metal content of model clay soils: total and their percentage in chemical soil fractions.

Metal	Total [mg/kg] Dry of Soil *	F_I_	F_II_	F_III_	F_IV_	Comments	Potentially Mobile Factions [%]	Non Mobile Factions [%]	M_f_	I_r_
		[%]								
BSvk										
Cu	6.28 ± 0.06	2.8	15.8	9.7	71.7	F_IV_ > F_II_ > F_III_ > F_I_	18.6	81.4	2.8	0.81
Cr	10.09 ± 0.11	1.9	13.3	21.9	62.9	F_IV_ > F_III_ > F_II_ > F_I_	15.2	84.8	1.9	0.79
Ni	7.22 ± 0.21	3.6	45.4	36.9	14.1	F_II_ > F_III_ > F_IV_ > F_I_	49	51	3.6	0.46
Pb	17.02 ± 0.01	9.2	24.5	27.1	39.2	F_IV_ > F_III_ > F_II_ > F_I_	33.7	66.3	9.2	0.61
Zn	64.54 ± 0.69	12.5	18.0	16.4	53.1	F_IV_ > F_II_ > F_III_ > F_I_	30.5	69.5	12.5	0.68
STx-1b										
Cu	8.97 ± 0.13	3.3	49.7	19.7	27.3	F_II_ > F_IV_ > F_III_ > F_I_	53	47	3.3	0.51
Cr	13.95 ± 0.34	0.1	3.7	52.3	42.9	F_III_ > F_IV_ > F_II_ > F_I_	3.8	96.2	0.1	0.73
Ni	7.57 ± 0.14	2.9	2.9	79.9	14.3	F_III_ > F_IV_ > F_II_ > F_I_	5.8	94.2	2.9	0.6
Pb	2.59 ± 0.04	0.2	5.3	37.9	56.6	F_IV_ > F_III_ > F_II_ > F_I_	5.5	94.5	0.2	0.79
Zn	73.68 ± 0.27	10.2	14.9	19.3	55.6	F_IV_ > F_III_ > F_II_ > F_I_	25.1	74.9	10.2	0.71
SWy-3										
Cu	12.80 ± 0.15	15.1	15.3	45.9	23.7	F_III_ > F_IV_ > F_II_ > F_I_	30.4	69.6	15.1	0.54
Cr	11.24 ± 0.13	75.7	1	13	10.3	F_I_ > F_IV_ > F_III_ > F_II_	75.8	24.2	75.7	0.23
Ni	7.27 ± 0.10	29	5.1	54.3	11.6	F_III_ > F_I_ > F_IV_ > F_II_	34.1	65.9	29	0.45
Pb	26.01 ± 0.05	37.2	13.7	39	10.1	F_III_ ≥ F_I_ > F_II_ > F_IV_	50.9	49.1	37.2	0.38
Zn	163.66 ± 1.50	13.5	19.1	21.4	45.9	F_IV_ > F_III_ > F_II_ > F_I_	32.6	67.4	13.5	0.64

* Metal content with standard deviation calculated for 4 samples using Grubbs’ statistical tests; F_I_—Exchangeable and weak acid soluble fraction, F_II_—Reducible fraction, F_III_—Oxidisable fraction, F_IV_—Residual fraction determined BCR method with using ICP-OES; Potentially mobile fractions—sum of F_I_ and F_II_; Non mobile fractions—sum of F_III_ and F_IV_; M_f_—mobility index; I_r_—stability index acc. to Formula (A1); Clays were obtained from the Source Clays Repository of The Clay Minerals Society and included STx-1b (a natural Ca-bentonite from Texas) and SWy-3 (a natural Na–Ca-bentonite from Wyoming), BSvk (a natural Ca-bentonite from Jelsovy Potok, Slovakia).

(A1) $$I_{r}=\cdot \frac{i^{2}\cdot F_{I}}{k^{2}}$$

where: *i*—the next step of sequential extraction; *k* = 4; *F_I_*—percentage of metal in the *i*-th chemical form.

**Table A2:** Metal content of model clay soils after contamination with Zn or Cu ions: total and their percentage in chemical soil fractions.

Metal	Total [mg/kg] Dry of Soil *	F_I_	F_II_	F_III_	F_IV_	Comments	Potentially/Mobile Factions	Non Mobile Factions	M_f_	I_r_
		[%]								
BSvk after saturation of 1 M ZnCl_2_^&^ (BSvk 1 M Zn)										
Cu	39.55 ± 0.25	8	50.8	22.7	18.5	F_II_ > F_III_ > F_IV_ > F_I_	58.8	41.2	8	0.44
Cr	87.25 ± 0.73	1.7	0.2	77.8	20.3	F_III_ > F_IV_ > F_I_ > F_II_	1.9	89.1	1.7	0.64
Ni	24.20 ± 0.21	10.6	6.5	54.3	28.6	F_III_ > F_IV_ > F_I_ > F_II_	17.1	86.9	10.6	0.61
Pb	17.50 ± 0.17	1.2	1.8	22.2	74.8	F_IV_ > F_III_ > F_II_ > F_I_	3	97	1.2	0.88
Zn	17,857 ± 89	31.7	24.1	31.8	12.4	F_III_ ≥ F_I_ > F_II_ > F_IV_	55.8	44.2	31.7	0.38
STx-1b after saturation of 1 M ZnCl_2_^&^ (STx-1b 1 M Zn)										
Cu	52.30 ± 0.53	3.2	2.4	8.3	86.1	F_IV_ > F_III_ > F_I_ > F_II_	5.6	94.4	3.2	0.92
Cr	82.40 ± 0.90	1.5	0.2	86.3	12	F_III_ > F_IV_ > F_I_ > F_II_	1.7	98.3	1.5	0.61
Ni	15.30 ± 0.11	13.4	7.5	58.9	0.2	F_III_ > F_I_ > F_II_ > F_IV_	20.9	79.1	13.4	0.36
Pb	7.53 ± 0.07	1	2.4	10.6	86	F_IV_ > F_III_ > F_II_ > F_I_	3.4	96.6	1	0.93
Zn	16,153 ± 75	27.3	22.6	29.4	20.7	F_III_ > F_I_ > F_II_ > F_IV_	49.9	50.1	27.3	0.45
SWy-3 after saturation of 1 M ZnCl_2_^&^ (SWy-3 1 M Zn)										
Cu	109.47 ± 1.34	26.9	3.2	9	60.9	F_IV_ > F_I_ > F_III_ > F_II_	30.1	69.9	26.9	0.68
Cr	114.85 ± 1.20	2.1	0.2	82.6	15.1	F_III_ > F_IV_ > F_I_ > F_II_	2.3	97.7	2.1	0.62
Ni	27.27 ± 0.23	8.7	4.7	45.7	40.9	F_III_ > F_IV_ > F_I_ > F_II_	13.4	86.6	8.7	0.68
Pb	14.61 ± 0.15	2.4	1	47.1	49.4	F_IV_ > F_III_ > F_I_ > F_II_	3.4	96.6	2.4	0.76
Zn	44,463 ± 124	34.9	26.7	28	10.4	F_I_ > F_III_ > F_II_ > F_IV_	61.6	38.4	34.9	0.35
BSvk after saturation of 1 M CuCl_2_^&^ (BSvk 1 M Cu)										
Cu	7677 ± 70	41.5	33.4	17.9	7.2	F_I_ > F_II_ > F_III_ > F_IV_	74.9	25.1	41.5	0.28
Cr	22.88 ± 0.17	0.6	45.9	19.0	34.5	F_II_ > F_IV_ > F_III_ > F_I_	46.5	53.5	0.6	0.57
Ni	22.02 ± 0.09	6	68.2	18.6	4.2	F_II_ > F_III_ > F_I_ > F_IV_	74.2	22.8	6	0.32
Pb	17.93 ± 0.18	1.6	25.7	14.9	57.8	F_IV_ > F_II_ > F_III_ > F_I_	27.3	72.7	1.6	0.73
Zn	95.61 ± 0.72	11.7	30.4	25.2	32.7	F_IV_ > F_II_ > F_III_ > F_I_	42.1	57.9	11.7	0.55
BSvk after saturation of 0.1 M CuCl_2_^&^ (BSvk 0.1 M Cu)										
Cu	23,284 ± 121	55.4	25.2	15.2	4.2	FI > F_II_ > F_III_ > F_IV_	80.6	19.4	55.4	0.23
Cr	13.97 ± 0.11	1.4	49.5	20	29.1	F_II_ > F_IV_ > F_III_ > F_I_	50.9	49.1	1.4	0.53
Ni	3.47 ± 0.13	5.1	39.9	35.4	19.6	F_II_ > F_III_ > F_IV_ > F_I_	45	55	5.1	0.5
Pb	22.90 ± 0.29	5.1	33.2	15.7	46	F_IV_ > F_II_ > F_III_ > F_I_	38.3	61.7	5.1	0.63
Zn	71.36 ± 2.13	10.1	24.1	10.1	55.7	F_IV_ > F_II_ > F_III_ > F_I_	34.2	65.8	10.1	0.68
BSvk after saturation of 0.25 M CuCl_2_^&^ (BSvk 0.25 M Cu)										
Cu	27,571 ± 90	21.9	46.9	26.7	4.5	F_II_ > F_III_ > F_I_ > F_IV_	68.8	31.2	21.9	0.33
Cr	35.73 ± 0.57	2.3	60.9	15.3	21.5	F_II_ > F_IV_ > F_III_ > F_I_	63.2	36.8	2.3	0.45
Ni	2.41 ± 0.65	3.2	38.2	32.4	26.1	F_II_ > F_III_ > F_IV_ > F_I_	41.4	58.5	3.2	0.54
Pb	14.53 ± 0.67	5.5	16.9	18.5	59.1	F_IV_ > F_III_ > F_II_ > F_I_	22.4	77.6	5.5	0.74
Zn	78.72 ± 0.86	15.8	28.9	16.3	39	F_IV_ > F_II_ > F_III_ > F_I_	44.7	55.3	15.8	0.56
BSvk after saturation of 0.5 M CuCl_2_^&^ (BSvk 0.5 M Cu)										
Cu	28,773 ± 162	12.4	64.3	15.4	7.9	F_II_ > F_III_ > F_I_ > F_IV_	76.7	23.3	12.4	0.33
Cr	58.09 ± 0.85	1	3.9	80	15.1	F_III_ > F_IV_ > F_II_ > F_I_	4.9	95.1	1	0.61
Ni	6.44 ± 0.40	6.3	10.1	61.3	22.3	F_III_ > F_IV_ > F_II_ > F_I_	16.4	83.6	6.3	0.6
Pb	13.31 ± 0.84	5.2	10.5	30	54.3	F_IV_ > F_III_ > F_II_ > F_I_	15.7	84.3	5.2	0.74
Zn	80.75 ± 2.03	10.5	22.5	17.8	49.2	F_IV_ > F_II_ > F_III_ > F_I_	33	67	10.5	0.65
STx-1b after saturation of 1 M CuCl_2_^&^ (STx-1b 1 M Cu)										
Cu	5427 ± 43	15.7	38	43.4	2.9	F_III_ > F_II_ > F_I_ > F_IV_	53.7	46.3	15.7	0.38
Cr	17.50 ± 0.30	0.3	3.8	87.4	8.5	F_III_ > F_IV_ > F_II_ > F_I_	4.1	95.9	0.3	0.59
Ni	16.27 ± 0.08	6.2	28	60.3	5.5	F_III_ > F_II_ > F_I_ > F_IV_	34.2	65.8	6.2	0.47
Pb	6.07 ± 0.10	2.5	8.6	39.9	49.1	F_IV_ > F_III_ > F_II_ > F_I_	11.1	89	2.5	0.74
Zn	92.96 ± 2.90	11.3	17.1	15.9	55.7	F_IV_ > F_II_ > F_III_ > F_I_	28.4	71.6	11.3	0.7
SWy-3 after saturation of 1 M CuCl_2_^&^ (SWy-3 1 M Cu)										
Cu	11,221 ± 61	13	42.3	43.1	1.6	F_II_ > F_III_ > F_I_ > F_IV_	55.3	44.7	13	0.37
Cr	38.88 ± 0.69	0.2	5.6	83	11.2	F_III_ > F_IV_ > F_II_ > F_I_	5.8	44.2	0.2	0.59
Ni	33.53 ± 0.52	6.1	38.9	51.8	3.2	F_III_ > F_II_ > F_I_ > F_IV_	46	54	6.1	0.42
Pb	9.50 ± 0.12	1.5	9.3	26.1	63.1	F_IV_ > F_III_ > F_II_ > F_I_	10.8	89.2	1.5	0.8
Zn	83.20 ± 1.30	8.3	14.2	10.2	67.3	F_IV_ > F_II_ > F_III_ > F_I_	22.5	77.5	8.3	0.77

^&^ Samples were rinsed from free chloride ions, * Metal content with standard deviation calculated for 4 samples using Grubbs’ statistical tests; F_I_—Exchangeable and weak acid soluble fraction, F_II_—Reducible fraction, F_III_—Oxidisable fraction, F_IV_—Residual fraction determined BCR method with using ICP-OES; Potentially mobile fractions—sum of F_I_ and F_II_; Non mobile fractions—sum of F_III_ and F_IV_; M_f_—mobility index; I_r_—stability index acc. to Formula (A1); Clays were obtained from the Source Clays Repository of The Clay Minerals Society and included STx-1b (a natural Ca-bentonite from Texas) and SWy-3 (a natural Na–Ca-bentonite from Wyoming), BSvk (a natural Ca-bentonite from Jelsovy Potok, Slovakia).

**Table A3:** Average unfrozen water content of Zn and Cu bentonites.

Soil	The average of the unfrozen water content [%] at a given temperature [°C] determined by ^1^H NMR method [26]								
	−32		−23	−14		−9	−5		−3
BSvk 1 M Zn	12.97 ± 0.43		15.15 ± 0.54	18.74 ± 0.29		24.29 ± 1.60	27.89 ± 1.21		33.55 ± 0.47
STx-1b 1 M Zn	15.17 ± 0.64		17.60 ± 0.54	20.38 ± 1.00		24.57 ± 0.10	28.11 ± 1.31		32.96 ± 1.37
SWy-3 1 M Zn	9.74 ± 0.62		11.69 ± 0.61	14.60 ± 1.12		17.73 ± 1.96	22.69 ± 1.93		30.79 ± 2.33
Soil	The average of the unfrozen water content [%] at a given temperature [°C] determined by DSC method [47]								
	−23	−20	−14	−12	−10	−8	−6	−4	−2
BSvk 1 M Cu	22.74 ± 1.65	22.98 ± 1.96	24.78 ± 2.05	25.91 ± 2.14	27.59 ± 2.08	30.43 ± 2.03	32.42 ± 2.21	37.31 ± 1.32	41.40 ± 4.07
BSvk 0.5 M Cu	22.47 ± 1.44	22.61 ± 1.65	24.16 ± 1.60	25.21 ± 1.33	26.21 ± 1.94	28.15 ± 1.51	31.86 ± 1.44	33.24 ± 1.13	41.79 ± 8.82
BSvk 0.25 M Cu	20.27 ± 1.18	20.27 ± 1.18	22.21 ± 0.67	22.99 ± 0.61	24.92 ± 0.47	27.68 ± 1.50	30.68 ± 2.01	33.95 ± 1.41	41.05 ± 6.61
BSvk 0.1 M Cu	17.51 ± 1.38	18.94 ± 1.50	20.03 ± 1.77	21.47 ± 1.41	23.61 ± 1.12	23.61 ± 1.12	24.65 ± 2.34	28.91 ± 0.73	32.26 ± 5.24

± Standard Deviation.
